# The impact of the South African Covid-19 lockdown on incidence and severity of traumatic brain injury at Tshepong hospital: A multivariate retrospective cohort study

**DOI:** 10.1016/j.heliyon.2023.e16906

**Published:** 2023-06-03

**Authors:** Jesse K.G. Bulabula, Akin B. Ogunrombi, Mxolisi B. Molefe, Victor Magumbeze, Kevin Y.G. Kumasamba

**Affiliations:** a Department of Neurosurgery, Faculty of Health Sciences, University of Cape Town, South Africa; b Department of Neurosurgery, Faculty of Health Sciences, University of Witwatersrand, South Africa; c Department of General Surgery, Faculty of Health Sciences, University of Witwatersrand, South Africa; d Department of Business Science, Faculty of Commerce, University of Cape Town, South Africa

**Keywords:** Covid-19 lockdown, Social restrictions, Traumatic brain injury, Social behaviour

## Abstract

**Background:**

Globally traumatic brain injuries (TBIs) are the leading cause of death in people under the age of 45. 2020 saw a series of social lockdowns as a response to the COVID-19 pandemic. We aimed to unveil the impact of the different levels of lockdown on TBI incidence at Tshepong Hospital.

**Method:**

A retrospective review of patients with TBIs during the first 30 days of each of the 5 lockdown levels, between 1st of April -20th October 2020 was conducted. Each lockdown level was compared to a control of a similar period in 2019.

**Results:**

Level 5 lockdown resulted in a 66% reduction in total incidence of TBI, with a decrease in the daily incidence median value to 0 when compared to its control group median of 1 (*P*-value 0.004). However, Level 3 and 2 resulted in a significant 133% and 200% increase respectively in TBI incidence for similar period the year before.

There was a 0,75% decrease in total trauma during the non-lockdown periods in relation to the lock down periods with a lockdown mean incidence of 53,4 (std Dev. 26.6) and non-lockdown mean of 53 (std Dev 20.8).

**Conclusion:**

The cumulative effect of the lockdowns made miniscule changes in the overall TBI incidence but led to significant variation in TBI incidence in the comparative months. A “rebound trauma” phenomena is observed in transitioning from severe social restrictions to milder ones with unemployment and unbanning of alcohol as possible contributary factors. Further studies are needed to investigate these complex interactions.

## Abbreviations list

TBI: Traumatic brain injury
COVID-19: Coronavirus disease 2019
SA: South Africa
MVA: Motor vehicle accident
GCS: Glasgow coma scale

## 1 Introduction

Traumatic brain injury (TBI) is defined as direct or indirect trauma to the brain which results in neurological symptoms or demonstration of intracranial pathology [1]. In South Africa (SA) trauma serves as the leading cause of death in children and young adults, where the rate of mortality from motor vehicle collisions and interpersonal violence is respectively five and four times the global average. Globally TBI is a significant cause of morbidity and mortality, with it being the leading cause of mortality in people under the age of 45 in the western hemisphere. In sub-Saharan Africa, the incidence of TBI is noticeably higher at 150–170/100 000 when compared to the global average of 106/100 000 [2].

In early December 2019 China recorded the beginning of an outbreak of coronavirus disease 2019 (COVID-19). It rapidly became a public health emergency of international concern with multiple countries implementing social restrictions in the form of lockdowns to prevent spread of the virus for fear of overwhelmed healthcare systems [3]. The South African Government subsequently developed series of lockdown strategies to adjust with the current state of the pandemic [4]. The lockdown aimed at placing limitations on interpersonal interactions and focusing on social distancing to prevent spread of the virus. Five different levels of lockdown were instated with level five having the most social restrictions and level one the least [[5], [6], [7], [8], [9]]. A summary of the lockdown restrictions is included in Supplementary Table S1.

As mentioned above, interpersonal violence and motor vehicle accidents (MVA's) serve as a major cause of TBI in SA. In addition, data exists reporting alcohol intoxication as present in one third to one half of hospitalizations in patients who suffer TBI with alcohol being a risk factor for severity [[10], [11], [12]]. It has therefore become necessary to investigate the effect of the COVID-19 lockdown with restrictions on locomotion and access to alcohol and whether the COVID-19 lockdown shows evidence for modifiable risk factors that impact TBI.

### 1.1 Aims/objective

To determine the effect of the different stages of the COVID-19 lockdown on Traumatic Brain Injury (TBI) at Tshepong Hospital. To be achieved by comparing the incidence, clinical severity (admission Glasgow coma scale (GCS)), radiological severity (Rotterdam score and Marshall classification) and prognosis (Glasgow outcome scale) of TBI during the different lockdown periods in 2020 to their respective periods in 2019.

### 1.2 Setting

Klerksdorp, situated in Northwest Province comprises of four sub-districts, falling under the Dr K Kaunda District (population of 725 364, with a population density of 49.5 people per km [2]), has an estimated medical scheme coverage of 23.7%, with interpersonal violence accounting for 60% of deaths between men aged 15 to 24^13^. Klerksdorp Tshepong Hospital complex (KTHC) is an 890 bedded tertiary hospital and acts as the primary drainage for the population of Matlosana (estimated 500000 people [13]).

## 2 Methodology

A retrospective study was done at Tshepong Hospital using the Picture archiving and communication system to collect all CT brains performed for suspected TBI during the different periods. Periods included in 2020 were 1st April – 30th April, 1st May - 30th May, 1st June - 30th June, 1st June - 30th June, 17th August 2020–15th September and 21st September – 20th October. Control periods included were the exact same dates mentioned prior in 2020 but in 2019. Files were then collected, and data points of interest were extrapolated. All patients who presented with signs and symptoms of a TBI who underwent a CT brain were included in this study. Patients who presented 14 days after an episode of trauma, required urgent surgery for non-neurosurgical trauma, had non-traumatic mass effect intracranial lesions and who sustained more than one-episode TBI less than 14 days apart were excluded from the study. Variables included were age, gender, daily TBI incidence, duration of admission, admission GCS, discharge GCS, diagnosis, polytrauma, mechanism of injury, Rotterdam score, Marshall classification, type of neurosurgical intervention and Glasgow outcome scale. Ethical approval was received from Patient Safety Group of Klerksdorp/Tshepong hospital complex and University of Witwatersrand ethics committee.

### 2.1 Statistical analysis

Statistical analyses were conducted with Microsoft Access, Excel and IBM SPSS Statistics [version 28 (IBM Corp., Armonk, New York, USA)]. The Shapiro–Wilk test was used to assess normal distribution of continuous data. For normally distributed data, a 1-way ANOVA followed by a Dunnett's post hoc test was conducted, whereas a Mann-Whitney *U* test and Kruskal–Wallis test with a Dunn's multiple comparisons test was used for non-parametric data. A confidence level of 95% (α:0.05) was enforced to show significance.

## 3 Results

A total of 615 patients who underwent CT scans for TBI were reviewed, of which 532 met the inclusion criteria. A Confidence interval of 95% and significance level of 0.050 was used for all tests.

**Fig. 1:**
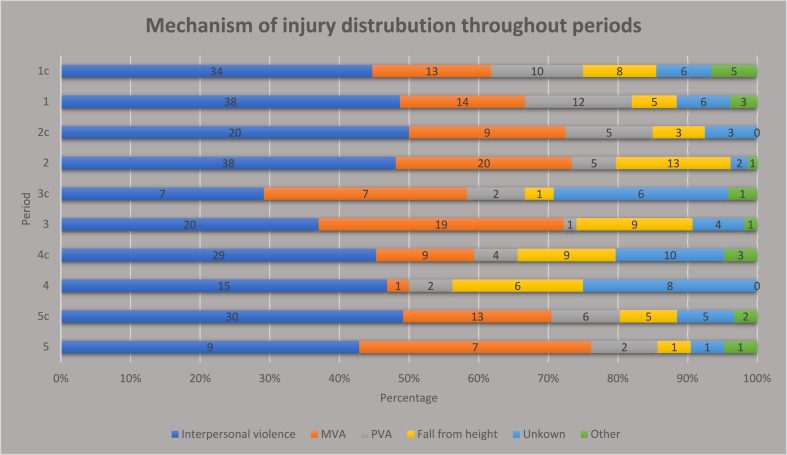
Graph showing distribution of each mechanism of injury as a percentage for each period. C (y –axis) denotes control for respective Lockdown level period.

The mean age was 30 years with a standard deviation of 15.93.426 patients (80%) were male and 106 were female (20%). Refer to Table 1 for further comparisons on sex during each lockdown level. Regarding mechanism of injury interpersonal violence was the most prominent (Fig. 1). The daily incidence rate of TBI differed across each lockdown level period compared to their respective control period except for lockdown level one. Refer to Table 2 for Mann Whitney- U details. During level 5 the total incidence of TBI was reduced to 21 with its respective control group incidence being 62. In level 4 incidence was halved at 32 compared to its control group with an incidence of 64. Level 3 incidence was increased at 56 in comparison to its control with an incidence of 24. Level 2 incidence doubled at 80 in relation to its control with an incidence of 40. During level 1 incidence was 76 which was similar to its control group's incidence of 78. The combined total number of TBI's during all 5 levels of lockdown was higher at 267 (mean of 53,4 std Dev. 26.6 and median of 56) when compared to the combined total of the 5 control groups which was 265 (mean 53, std Dev 20.8 of and median of 61). The distribution of the incidences amongst the 5 lockdown periods was the same in comparison to the incidences of the control periods (P value 0.98 with a T value of 0.027). Refer to Table 2 for details.

**Table 1:** Mean age and gender comparison throughout all lockdown levels and their control periods.

Lockdown level	Mean age (standard deviation)	Control group Mean age, standard deviation	Male/Female percentage of TBI's	Control group male/Female percentage of TBI's
5	29.76 (15.47)	29.52 (17.21)	90/10	79/21
4	24.7 (17.5)	26.91 (15.49)	84/16	90/10
3	30.68 (15.6)	35.38 (16.34)	80/20	75/25
2	28.75 (14.88)	32.95 (13.98)	85/15	88/12
1	32.17 (15.59)	30.54 (16.82)	87/13	63/37

**Table 2:** Summary of results comparing incidence across 30-day lockdown periods and their respective control groups.

Lockdown level	Total incidence	Total incidence for control group	Total incidence Percentage difference from control group	Daily incidence median value (IQ range)	Daily incidence median value for control group (IQ range)	Mann-Whitney U value for daily incidence	P value
5	21	61	66% decrease	0 (1)	1 (2)	636.5	0.004
4	32	64	50% decrease	1 (1)	2 (2)	614.0	0.011
3	56	24	133% increase	2 (3)	1 (1)	283.0	0.010
2	80	40	200% increase	2.5 (2)	1 (1)	261.0	0.004
1	78	76	2,5% decrease	2 (2)	2 (2)	430.0	0.781

Table shows the results of the Mann-Whitney U results assessing for a significant difference in daily incidence rate for each lockdown period and their control period. IQ= Interquartile range.

The Marshall classification was the same across all the different periods except lockdown level 1 and the control for lockdown level 2 (P value of 0.034). Refer to Table 3 for comparative statistics. The Rotterdam score was the same across all the different periods (P > 0.05) with a median score of 2 and interquartile range of 0 for all time periods.

**Table 3:** Results of the Kruskal Wallis test assessing for significance between the Marshall Classification per patient for each lockdown level and their control group.

Lockdown level	Marshall classification Median (interquartile range)	Control group Marshall classification Median (interquartile range)	Adjusted significance value
5	1 (1)	1 (1)	1.00
4	1 (1)	1 (1)	1.000
3	1 (0)	1 (1)	1.000
2	1 (1)	1 (0)	.691
1	1 (1)	1 (1)	.486

Asymptotic significances (2-sided tests) are displayed. The significance level is 0.050. Significance values have been adjusted by the Bonferroni correction for multiple tests.

Due to incomplete data sets (Table 4), 389 of the 532 cases were used when comparing presenting GCS across all period groups with no statistically significant difference found (P > 0.05) with a median GCS of 15 throughout all periods. Regarding the GCS at presentation, all periods had an interquartile range of 0 except lockdown level 5 with an interquartile range of 1. Incomplete data sets resulted in 384 of the 532 cases being used to compare the Glasgow Outcome Scale across all periods which also showed no difference (P > 0.05) and a median of 5.

**Table 4:** Table showing number of files with incomplete datasets for each lockdown period.

Period	Patients	Patients with incomplete data sets	Percent of files with incomplete datasets within period
5	21	4	19
5c	61	17	28
4	32	12	38
4c	64	17	27
3	56	12	21
3c	24	8	33
2	80	19	24
2c	40	13	33
1	78	22	28
1c	76	19	25

C denotes lockdown level control group.

Of 384 patients with complete data sets, 155 were admitted with a median admission of 0 patients per period. There was no difference between admission percentage between different periods (P Value 0.213). Admission duration was not significant across categories of period (P > 0.05) with a mean admission duration of 1,5 days (interquartile range of 1) and a median admission duration of 0 days, refer to Table 5 for further details. Of the 384 patients with complete data sets, 51 were polytrauma. 13% of patients presenting with TBI had polytrauma with no difference in incidence of polytrauma associated TBI's amongst different periods (P > 0.05).

**Table 5:** Number of admissions per lockdown period and median admission duration for each lockdown period and their respective control groups.

Period	Number of admissions (Percent)	Number of admissions (Percent) For control group	Median admission duration (Interquartile range)	Median admission duration (Interquartile range) for control group
5	5 (31)	15 (34)	0 (1)	0 (1)
4	11 (55)	13 (28)	1 (2)	0 (1)
3	19 (43)	5 (31)	0 (2)	0 (2)
2	21 (34)	7 (26)	0 (1)	0 (1)
1	29 (51)	24 (42)	1 (3)	0 (2)

## 4 Discussion

The introduction of regulated lockdowns serves a social experiment, giving us the opportunity to better understand the impact of social restrictions on trauma and subsequent TBI. This study is one of the first studies which looks in greater detail, the terms of social restrictions in each lockdown stage and the impact it has on TBI.

Level 5 lockdown resulted in a 66% reduction in total incidence of TBI, with a decrease in the daily incidence median value to 0 compared to its control group median of 1 (P value 0.004). This is expected in level 5 as citizens were not allowed to leave their households except for gaining access to essential services and necessities with all social gatherings being banned, thus, limiting the amount of interpersonal violence and MVA's which account for majority TBI's (Fig. 1).

A similar reduction in level 4 lockdown is noted with a 50% reduction in total incidence with a decrease in daily median to 1 from a control group median of 2 (P value 0.011). The lifting of the 24-h curfew to a 9-h one (20h00 to 05h00) curfew, allowing people to exercise 5 km from their households and permission of funerals with attendees less than 50 people were introduced in the level 4 lockdown. This possibly explains the slight increase in incidence from level 5 but still an overall significant decline in TBI due to the hard lockdown. Multiple studies looking at effect of lockdowns on trauma throughout the world report similar findings as what was evidenced in level 5 and 4 lockdowns. A systematic review done on global orthopaedic injuries secondary to trauma saw a decline in trauma ranging from 20.3% to 84.6% with interpersonal violence being the leading cause and a significant reduction in MVA's [14].

Level 3 lockdown unexpectantly resulted in a disproportional 133% increase in TBI incidence with an increase in daily incidence median to 2 from a control group median of 1 (P Value 0.01). The major changes in level 3 were in the form of allowance of locomotion and opening of more businesses (except liquor and entertainment services). The percentage of MVA's were highest during level 3 lockdown which is expected, as June 2020 saw an increase of 19% from May 2020 with regards to vehicle usage for South Africans according to tracker database from 2020 [15]. This coincides with the increase in percentage of MVA's from lockdown level 4 to 3 which provides a strong argument for increased locomotion being responsible for increased incidence of TBI from level 4 to 3.

However, this doesn't fully explain the observed significant increase of 133% when compared to the control group for level 3. Traffic reports from 6 major cities in South Africa showed an overall decline in congestion in 2020 as compared to 2019 [16]. One plausible explanation for the 133% increase is that June marks the beginning of winter in the northwest with some of its lowest temperatures [17]. Studies have shown that winter periods result in a decrease in overall trauma [18]. However, this is negated by the fact that Northwest experienced a colder June in 2020 relative to 2019 [19,20]. This leads to a possible theory that the lifting of locomotion restrictions with opening of more businesses led to a ‘‘rebound” influx of movement, resulting in an increased number of MVA's and overall TBI incidence which correlates with the 171% increase in MVA's (Fig. 1) resulting in increased TBI's during lockdown period 3 compared to its control period in June 2019.

Lockdown level period 2 incurred another “rebound phenomena” with a twofold increase in TBI incidence with an increase in daily TBI incidence median to 2.5 from 1 compared to its control group (p value 0.004), which was the highest incidence throughout all the periods. Key changes in lockdown level 2 where in the form of a reduced curfew of 6 h (22h00 – 4h00), unrestricted movement between provinces, the permission of social gatherings (limited to 50 people), opening all institutions (including entertainment and bars) and the more controversial lifting of the liquor ban. The main factor for this surge in trauma is likely to be due to the permission of alcohol intake. South Africa is infamous for its alcohol consumption culture, being ranked 5th in 2016 per capita per year in the world, with 59% of their drinkers being classified as heavy or binge drinkers, consuming 60 g or more of pure alcohol on at least one occasion over a 30-day period. Having 40% of road traffic deaths which involve half of the pedestrians containing a blood-alcohol level above the legal limit for driving [21,22]. A paper published in 2003 revealed that between 35.8% and 78.9% of patients in trauma units in major cities in South Africa tested positive for alcohol, and those who tested positive for alcohol were more likely to have interpersonal violence as the cause of their injuries compared to motor vehicle accidents and other unintentional injuries [23]. Another paper also noted interpersonal violence being the leading determinant of death in alcohol related mortality with alcohol harm accounting for an estimated 7.1% of all deaths and 7.0% total disability-adjusted life years in 2000 [24].

Another factor that might have contributed to the rise in TBI's, is lockdown level 2 coinciding with the second quarter of the financial year which saw a record low employment rate of 36% which was previously 42% during the first quarter of the financial year with a 9,9% drop in the Northwest in particular [25]. A study in America looking at the relationship between trauma admission rates during the Great recession of 2008–2010 indicates trauma admission rates decreased in neighbourhoods with the highest unemployment levels but increased during the same period in neighbourhoods with lower unemployment [26]. Another study in Malawi also inferred a strong correlation between unemployment and interpersonal violence, particularly amongst those between the age of 25 and 45^28^. Overwhelming evidence indicates that nationwide lockdowns lead to significant increments in the prevalence of mental health issues in the form of anxiety, depression, and psychological distress [27,28]. This brings in another possible factor that in the prolonged hard lockdowns where mental health issues accumulate then followed by alleviation of social restrictions, avenues are created for individuals to exaggerate their social interaction to help mitigate their mental health. This surge of social interaction especially in the setting of alcohol increases the chance of one being exposed to trauma and subsequently TBI. Considering all the above mentioned, the significant rebound in TBI incidence during level 2 is most likely due to South Africa's problematic binge drinking culture precipitated by a substantial increase in unemployment in the setting of the psychological effects of transitioning between lockdowns levels.

Lockdown level 1 indicated a 2,5% decrease in TBI incidence with a daily TBI incidence median of 2, equal to its control period. Level 1 lockdown saw the opening of all businesses except night clubs, a curfew of 4 h (midnight to 04h00), easing of the alcohol limitations, authorisation of international travel and increasing the number of participants permitted at gatherings. These changes essentially made no difference on TBI incidence.

When analysing the total incidence of TBI between the non-lockdown and lockdown periods, there was a 0,75% decrease in trauma during the non-lockdown periods in relation to the lock down periods with a lockdown mean incidence of 53,4 (std Dev. 26.6) and non-lockdown mean of 53 (std Dev 20.8). It is evident that the implementation of the lockdown changed the distribution of TBI incidence throughout each period however had minimal effect on the total incidence throughout the cumulative lockdown periods when compared to the cumulative control periods.

There were no other differences noted between the lockdown periods and their respective control groups in terms of other parameters. The mean age was 30 (Standard deviation- 15.93) throughout all periods with the means for each period falling within that standard deviation. There was no obvious pattern with age differences in lockdown and pre lockdown control groups with the greatest difference in mean being present in lockdown level 3 (Table 1). Proportion of different sex groups affected in pre-lockdown and lockdown, followed a similar ratio of the overall percentage distribution (80% male and 20% female). The biggest change in proportion was in lockdown level one where 87% of males were affected and 13% of females of the total patients in that period compared to 63% of males and 37% of females in the control group (Table 1).

Regarding radiological severity for the patients in each lockdown period, no significant differences were found between the lockdown period and their control groups. A median Marshall classification of 1 and median Rotterdam score of 2 was present throughout all periods, implicating no difference in severity for all lockdown levels. The median GCS was 15 throughout all periods which was in keeping with radiological severity alluding to the assumption that severity of TBI was not affected by the different lockdown levels.

## 5 Conclusion

The effects of the lockdown were noted mainly in TBI incidence. Despite an overall similar incidence in TBI, there was great variation throughout the different periods. Level 5 and 4 were effective in reducing TBI incidence yet were not economically sustainable leading to a decline in employment. The sudden lifting of travel restrictions precipitated a surge in MVA related TBI during level 3 with a “rebound” effect. The social and psychological aftereffects of level 5 and 4 coupled with alleviation of alcohol restrictions may have manifested in level 2 as a second rebound in overall TBI incidence, negating the reduction in TBI in previous levels of lockdown and level 1 lockdown had almost no impact. It is unclear however if these findings could be extrapolated to trauma in general or TBI specifically. This study also highlights the complex and often unpredictable interactions between social constructs and disease. Further studies are needed to unravel these relationships. This brings awareness to the need for preparation of shifting resources to Trauma units in anticipation of shifting from a highly restrictive lockdown to a less restrictive one and the importance of being cognisant of the rebound socioeconomic consequences of different lockdown restrictions.

## 6 Limitations

This study was restricted by the minimum duration of lockdown periods which limited the sample size of period days to 30. Another limitation was due to this study only being conducted at one hospital and more hospitals would provide a greater patient pool to be more representative of the entire country of South Africa. Up to one third of patients from each period had incomplete data sets making it difficult to definitively assess and comment on hospital course, prognosis via Glasgow Outcome scale and clinical severity via GCS. This study reflects the effects of the specific South African lock downs imposed during 2020, presumedly making it less applicable to other lockdowns from other countries.

## Funding

This research did not receive any specific grant from funding agencies in the public, commercial, or not-for-profit sectors.

## Author contribution statement

Jesse George Kwete Bulabula: Conceived and designed the experiments; Performed the experiments; Analyzed and interpreted the data; Contributed reagents, materials, analysis tools or data; Wrote the paper. Akin. B Ogunrombi: Conceived and designed the experiments; Contributed reagents, materials, analysis tools or data. Mxolisi. Brilliant Molefe: Contributed reagents, materials, analysis tools or data. Victor Magumbeze: Conceived and designed the experiments. Kevin. Y.G Kumasamba: Analyzed and interpreted the data.

## Data availability statement

Data will be made available on request.

## Declaration of interest's statement

The authors declare no conflict of interest.

## Declaration of competing interest

The authors declare that they have no known competing financial interests or personal relationships that could have appeared to influence the work reported in this paper.

## Appendix A Supplementary data

supplementary table 1_V2.docx supplementary table 1_V2.docx
