# Supplementary material for: The impact of the South African Covid-19 lockdown on incidence and severity of traumatic brain injury at Tshepong hospital: A multivariate retrospective cohort study
Source: Heliyon. 2023 Jun 3;9(6):e16906. doi: 10.1016/j.heliyon.2023.e16906 (PMC10239285; doi:10.1016/j.heliyon.2023.e16906)
Supplement: supplementary table 1_V2.docx [file mmc1.docx]

| Supplementary table 1. Summary of South African lockdown periods used during study with their respective restrictions. | | | | | | |
| --- | --- | --- | --- | --- | --- | --- |
| Time period | Period | Liquor ban | Curfew | Movement | Gatherings | Places Open to the public |
| 1/4/2019  -30/4/2019 | Control for lock down level 5 | None | None | Unrestricted | Unrestricted | All |
| 1/5/2019  -30/5/2019 | Control for lock down level 4 | None | None | Unrestricted | Unrestricted | All |
| 1/6/2019  -30/6/2019 | Control for lock down level 3 | None | None | Unrestricted | Unrestricted | All |
| 17/8/2019  -15/9/2019 | Control for lock down level 2 | None | None | Unrestricted | Unrestricted | All |
| 1/4/2020  –30/4/2020 | Lock down level 5 | Present | 24/7 | Restricted between provinces, metropolitan and district except for Essential workers. | All Banned | All essential services to buy necessities like food and medicine. |
| 1/5/2020  -30/5/2020 | Lock down level 4.  Once off return to place of residence (1/5/2020 to 7/5/2020) | Present | 20:00 until 05:00 | Restriction between provinces.  Exercise within 5 km of their place of residence (06:00 and 09:00).  Allowed for certain workers and essential workers. | All Banned except for  Funerals (50 people max), Workplace  and buying or obtaining essential goods and services. | All essential services to buy necessities like food and medicine. |
| 1/6/2020  -30/6/2020 | Lock down level 3 | Present | 21:00 until 04:00 | Restriction between provinces.  Exercise within 5 km of their place of residence (06:00 and 18:00)  Allowed for certain workers and essential workers | Banned, except for  Level 4 exceptions, attending faith-based institutions, agricultural auctions, professional non-contact sports matches and auctions. | All businesses except for  liquor and tobacco retailers, short-term home rental for leisure purposes and entertainment activities. |
| 17/8/2020  -15/9/2020 | Lock down level 2 | On-site consumption will be permitted until 22h00, while off site consumption will be allowed from Monday to Thursday between 09h00 – 22h00. | 22h00 – 04h00 | Unrestricted except for International travel. | Gatherings of more than 50 people are still prohibited, as are sports events with spectators | All businesses with re-opening of bars and liquor retailers. |
| 21/9/2020  -20/10/2020 | Lock down Level 1 | Licensed liquor to be sold. Off-site consumption permitted from 09h00 to 17h00, Mondays to Fridays, excluding weekends and public holidays; and  by a licensed premise for on -site consumption is permitted | 00h00  -  04h00 | Unrestricted with exceptions for international travel for countries with high COVID infection/transmission rates. | Attendance at a funeral is limited to 100 persons or less: Provided that not more than 50 percent of the capacity of the venue is used.  Gatherings at social events are limited to 250 persons or less in case of an indoor gathering and 500 persons or less in case of an outdoor gathering (provided 50% or less max capacity with social distancing.). | All businesses except night clubs are closed to the public. |
